# Audio-Vestibular Evaluation of Pediatric Pseudo-Conductive Hearing Loss: Third Window Syndromes

**DOI:** 10.3390/audiolres14050066

**Published:** 2024-09-06

**Authors:** Gorkem Ertugrul, Aycan Comert, Aysenur Aykul Yagcioglu

**Affiliations:** 1 Department of Audiology, Faculty of Health Sciences, Hacettepe University, 06230 Ankara, Turkey; 2 Department of Audiology, Faculty of Health Sciences, KTO Karatay University, 42020 Konya, Turkey

**Keywords:** third window, semicircular canal dehiscence, enlarged vestibular aqueduct, perilymphatic fistula, pseudo-conductive hearing loss, pediatric

## Abstract

Conductive hearing loss caused by external or middle ear problems prevents the transmission of sound waves from the external auditory canal to the cochlea, and it is a common condition, especially in pediatric patients aged 1–5 years. The most common etiological factors are otitis media and cerumen during childhood. In some patients, external and middle ear functions and structures may be normal bilaterally despite the air-bone gap on the audiogram. This condition, which is often a missed diagnosis in children, is defined as a pseudo-conductive hearing loss (PCHL) caused by third window syndromes (TWSs) such as semicircular canal dehiscence, inner ear malformations with third window effect, and perilymphatic fistula. In this review of the literature, the authors emphasize the pitfalls of pediatric audio-vestibular evaluation on TWSs as well as the key aspects of this evaluation for the differential diagnosis of PCHL brought on by TWSs. This literature review will provide audiologists and otologists with early diagnostic guidance for TWSs in pediatric patients.

## 1. Introduction

The inability of sound waves to pass from the external auditory canal to the cochlea is known as conductive hearing loss (CHL), and it is brought on by issues with the middle or outer ears. This condition is particularly common in pediatric patients aged 1–5 years. The most common etiological factors during childhood are otitis media and cerumen [1,2]. An air-bone-gap (ABG) suggesting a conductive hearing loss can still be observed in pure tone audiometry in the presence of normal external and middle ear. A third window, which is a bony defect that arises as a connection between the middle and inner ear or between the inner ear and the cranial cavity, can also generate pseudo-conductive hearing loss (PCHL) due to the so-called third window effect. The cochlea has two windows that are referred to as round and oval windows, respectively. The inner ear appears to have a third window outside of these two windows due to a low-frequency ABG and PCHL energy through a third window [3]. Similarly, the cochlear travelling wave extends from the oval window to a pathological third window at very low pressure, which enhances or decreases bone conduction thresholds and increases the ABG. PCHL, which is often a missed diagnosis in children, is caused by third window syndromes (TWSs), including enlarged vestibular aqueducts (EVAs), incomplete partition Type-II (IP-II), semicircular canal dehiscence (SCD), and perilymphatic fistulas (PLFs) [3,4].

This review of the literature covers pediatric audio–vestibular examination for PCHL caused by TWSs, specifically SCD, EVAs, and PLFs.

## 2. Etiological Factors of Pseudo-Conductive Hearing Loss in Children

According to Dasgupta et al. [5], there are many other third window effects, including new descriptions of TWSs, although most studies on TWSs concentrate on superior SCD, and in adults, they are focused on (1) posterior and lateral SCD; (2) cochlea–facial nerve dehiscence; (3) cochlea–internal carotid artery dehiscence; (4) cochlea–internal auditory canal dehiscence; (5) X-linked gusher syndrome; (6) perilymph fistulas; (7) facial nerve canal dehiscence; (8) wide vestibular aqueduct in children; (9) post-traumatic hypermobile stapes footplate; (10) otosclerosis with internal auditory canal involvement; (11) bone dyscrasias, for example, Paget’s disease of the bone and osteogenesis imperfecta; and (12) endolymphatic hydrops [4,6,7]. In this literature review, the authors focused on SCD, EVAs and PLFs as the causes behind TWSs.

### 2.1. Inner Ear Malformations Causing the Third Window Effect

Inner ear malformations (IEMs) account for approximately 20% of congenital hearing loss cases [8]. In this literature review, the authors only focused on the most common types of IEM-causing TWSs in children: IP-II and EVAs.

IP-II and EVAs are well known for causing the third window effect in the inner ear [9,10]. IP-II can be seen in isolation or combination with EVAs and vice versa [11,12,13,14]. A combination of nonsyndromic EVAs and Mondini deformity was found in 71% and 29%, respectively, of 66 children over the age of 16 in a retrospective analysis [15]. Mondini deformity is the term used to describe IP-II, which coexists with a dilated vestibule and EVA [13]. The incidence of EVAs in pediatric patients ranges from 5% to 15% [16,17]. IP-II and EVAs can cause progressive or sudden, pseudo-conductive, sensorineural, or mixed hearing loss [18,19]. The auditory and vestibular systems may be affected by EVAs. Notably, mixed hearing loss, which is often a late-onset condition (diagnosis in adolescence or adulthood), has been diagnosed in patients with EVAs [20]. Progressive SNHL, whose reported prevalence varies from 18% to 65%, is commonly linked to EVAs [17,21]. Hearing loss may not occur or be obvious from birth, even though an EVA is a congenital IEM. Many children with EVAs demonstrate a postlingual onset of deafness [12]. Therefore, these patients should avoid head trauma as a trigger of increased CSF pressure, which can pass into the inner ear and cause sudden or progressive hearing loss.

An explanation based on the impact of inner ear fluids on inner ear structures can elucidate the connection between the enlargement of the vestibular aqueduct and the various characteristics of hearing loss (severity, onset, and type). EVAs can result in increased inner ear pressure and electrolytic imbalance of inner ear fluids, which can lead to hearing loss and vestibular dysfunction. The conductive component of the hearing loss can also be brought on by high inner ear fluid pressure, which restricts stapes movement. The third window effect of EVAs results in an ABG resembling that of SCD [15,21].

### 2.2. Semicircular Canal Dehiscence

Located around the membranous semicircular canal, SCD is a pathological opening in the bony otic capsule. This causes the membranous labyrinth to transmit pressure abnormally both to and from it. In the labyrinth, it provides a third mobile window in addition to the round and oval windows. It was first diagnosed in 1998 by Minor et al. [22], who examined the temporal bone computed tomography (CT) results of eight patients with vertigo caused by sound and pressure applied to the external auditory canal [23]. In a study conducted in children aged 2 years, the prevalence of posterior SCD was 20%, and that of superior SCD was 13.8% [24]. In a different study examining the temporal bone CT results of 228 children aged 7 years, the prevalence of posterior SCD was 16.7%, and the prevalence of superior SCD was 11.9% in children younger than six months [23]. It has been stated that these rates decrease with age due to the thickening of the bones, and the situation reverses after one year of age [24]. One of the reasons for this is that radiological imaging methods are difficult to apply in children. Another reason is the diversity of methodological approaches. SCD has been classified as “dehiscent”, “possibly dehiscent”, or “thinned bone” by the authors of several publications [24,25]. The prevalence of dehiscence in the pediatric population was greater when all groups were combined. 

There are two theories for the mechanism of SCD: congenital and acquired. According to congenital theory, semicircular canals begin with the formation of the membranous labyrinth from the otocyst. The semicircular canals develop in the following sequence: first, the superior canal, followed by the posterior canal, and finally the horizontal canal [26,27]. When the membranous labyrinth reaches adult size, ossification begins and then progresses to the semicircular canals in the order previously listed. Pressure and fluid changes originating from the dura and dural sinuses in the middle and posterior cranial fossa can cause dehiscence [26,28].

### 2.3. Perilymphatic Fistula

A PLF results from an improper perilymph connection between the middle and inner ear due to an otic capsule defect. This abnormal connection in the PLF decreases the impedance of the vestibular fluid and causes symptoms such as sensorineural hearing loss (SNHL), tinnitus, auditory fullness, imbalance, motion intolerance, and positional dizziness [29]. Numerous etiological causes can lead to PLFs, including middle ear surgery, stapedectomy, tumors, cholesteatoma, head trauma, cough, acoustic trauma, and temporal bone fractures [30,31]. The onset of symptoms may occur weeks, months, or even years after a triggering event, and the symptoms experienced by the patient may vary [32]. Post-traumatic hearing loss in PLFs is sudden or progressive. Dizziness is typically manifested as severe vertigo, imbalance, or positional dizziness. The severity of hearing loss and dizziness may be related to the severity of the trauma [33].

## 3. Clinical Characteristics of Third Window Syndromes

The prevalence of the TWSs is quite rare (<1%) in children [5]. Older children may describe symptoms resulting from the third window, such as autophony, gaze-evoked tinnitus (audible eye movement), pulsatile tinnitus, Tullio phenomenon, Hennebert’s sign, and conductive dysacusis (such as hearing one’s footfall) [5,34]. As in adults, children with TWSs often exhibit low thresholds and amplitude asymmetry on vestibular evoked myogenic potential (VEMP) tests and an ABG at low frequencies on the audiogram, even in the presence of normal tympanometry [5,35]. Dasgupta et al. [5]. found that 87.5% of the patients in a study investigating audio-vestibular findings in TWSs in children showed conductive and mixed-type hearing loss with air-bone gap despite normal tympanometry findings. They reported that 50% of these patients experienced transitory otoacoustic emissions [5].

SCD is characterized by sound and pressure-induced vertigo or nystagmus, aural fullness, autophony, pulsatile tinnitus, and hyperacusis, and PCHL is often seen at low frequencies (<2000 Hz). In a study conducted in the pediatric population, it was found to be an auditory-to-vestibular sign and a symptom ratio of 4:1 [36]. The sensitivity and specificity of air conduction (AC) cervical VEMP (cVEMP) to identify TWSs have been reported to exceed 90% [37]. In a SCD cohort with a wide age range, Castellucci and colleagues [38] demonstrated that the size of the SCD affects the ABG, AC pure tone average (PTA), and the amplitudes and thresholds of both cVEMP and ocular VEMP (oVEMP). The researchers demonstrated a positive correlation between the size of the SCD and audio-vestibular findings (AC PTA, ABG, bone conduction (BC) cVEMP amplitude, and AC and BC oVEMP amplitude). In contrast, compared to SCD in the ampullary arm and superior petrosal sinus, SCD location in the arcuate eminence was negatively correlated with SCD length, AC oVEMP, cVEMP thresholds, and superior canal vestibulo-ocular reflex (VOR) gain [39]. The vHIT in SCD also may show abnormalities gain [40]. This is likely due to the observation that in the pediatric population, the vestibular system responds to a third window. The possible explanations for the reduced VOR gain during the vHIT test in patients with SCD include two mechanisms: (1) incomplete canal plugging and (2) loss of mechanical energy [38]. As in adults with TWSs, VEMP thresholds are predicted to decrease and amplitudes are believed to increase in children [40,41]. Manzari et al. [42] claimed that the presence of AC or BC oVEMP at 4000 Hz can be used to rapidly diagnose SCD in adults. Although Wiener-Vacher et al. [43] reported normative values of BC and AC cVEMP in healthy children, there are no specific VEMP parameters established yet for diagnosing SCD in children. VEMP procedures recommended for SCD in adults may also apply to children.

One known cause of vestibular dysfunction is EVA/IP-2 [44,45]. Zalewski et al. [44] described at least one vestibular symptom in 45% (48/106) of patients with EVAs (mean age 12 ± 11.5 years). The most common vestibular symptoms were rotatory vertigo (23%), head tilt, vomiting at prelingual age (20%), and clumsiness (18%) [44]. Consistent with the findings of Zalewski et al. [41], recent studies have indicated that some children with EVAs/IP-II (10–37%) had delayed gross motor function [45,46]. In a recent study [46], 13.9% of children with IP-2 and EVAs experienced vestibular loss on the non-implanted side, and this rate increased to 19% on the CI side. Although hearing loss in Mondini deformity is usually sensory in origin, audiograms in these individuals sometimes indicate PCHL [47]. This is attributed to low compliance between the perilymphatic and CSF compartments, resulting in the better-than-normal bone conduction thresholds sometimes observed in these malformations, which is similar to the third window effect [47]. In Mondini deformity, as in PLFs or SCD, patients may experience dizziness or the Tullio phenomenon caused by loud sounds [47]. In addition, patients often experience vestibular problems, such as vertigo, motor delay, and imbalance [48]. According to the study of Karlberg and colleagues [9], a female patient with CHL believed to be due to otosclerosis was diagnosed with Mondini deformity based on audio-vestibular evaluations and radiological results. The CHL in this patient was caused by the pathological third window resulting from inner ear dysplasia. The authors suggested that Mondini dysplasia should be added to the causes of PCHL in the inner ear [9]. A recent study reported that third window anomalies and congenital IEMs may accompany each other in children with conductive hearing loss [10]. It should be known that IEMs such as Mondini deformity may be among the causes of CHL in pediatric patients. Nevertheless, additional research is required to assess the underlying pathophysiology of various forms of hearing loss associated with third window anomalies and IEMs.

There are only a few studies on PLFs in children. In a study of 37 children with congenital PLFs, 28 (76%) of the children had a history of middle ear disease. In particular, in the pediatric population, when sensorineural hearing loss develops or progresses after otitis media attacks, the possibility of congenital PLFs should be considered [49]. In another study in which 28 pediatric patients experienced dizziness after a concussion, two children with peripheral vestibular disorder had PLFs [50]. In a study examining children with perilymphatic fistulas [48], 6 of 16 patients (37.5%) reported that the age of onset of symptoms was under 2 years old. Complaints of intermittent dizziness or observed imbalance seizures (56%) accompanying hearing loss in children indicate a PLF [51]. Therefore, the possibility of congenital PLFs should be considered in all infants and children with progressive, fluctuating, or sudden hearing loss. PCHL and positional nystagmus at low frequencies may be observed due to the third window lesion in PLFs. TWSs may occur with symptoms similar to those of PLFs or may occur together with PLFs [32]. In the differential diagnosis of TWSs and PLFs, clinicians should focus on suspicion of PLFs if patients with nonspecific audio-vestibular symptoms do not respond to conventional medical treatments or vestibular rehabilitation, and there is a history of post-traumatic onset [52,53].

In terms of the published evidence, audio-vestibular evaluation of PCHL caused by TWSs and findings are summarized in Table 1.

## 4. Pitfalls of Pediatric Audio-Vestibular Evaluation on Third Window Syndromes

Otitis media with effusion, a common middle ear pathology in children, often masks the TWS phenotype. This is one of the most important issues and leads to a TWS being missed. Another significant factor contributing to the underdiagnosis of TWSs in children is the discovery of normal tympanograms even in cases in which hearing loss is present but not exhibiting any symptoms. In these pediatric cases, evaluating AC pure tone hearing thresholds alone may result in the missed diagnosis of TWSs causing PCHL in children. It has been proposed that TWSs can be diagnosed based on the existence of negative BC thresholds [55]. Nonetheless, Merchant et al. [56] stated that the ABG should be taken into consideration as a diagnostic criterion.

Auditory brainstem response (ABR) testing is a crucial diagnostic tool for confirming hearing loss in pediatric patients. When BC hearing thresholds cannot be assessed or are not reliably obtained in young children, BC ABR testing is recommended to determine the type of hearing loss [57]. Our clinical experience suggests that BC hearing thresholds and BC ABR can be used to detect an ABG, an important audiological indicator of TWSs in young children. Moreover, the presence of otoacoustic emission responses despite PCHL and a low-frequency air-bone gap with supranormal BC thresholds are indicators of TWSs [4,5].

On the other hand, it is not possible to differentiate the third window effect in children with only the ABG as determined by audiological evaluation. Pediatric vestibular evaluation is required. Vestibular complaints in children may not be as clear as those in adults or may not be fully expressed by children. Therefore, after spontaneous nystagmus, oculomotor, and positional tests, it is important to rule out the third window effect by using oVEMP (elevated amplitudes), cVEMP (decreased thresholds) and vHIT (reduced VOR gain and presence of saccades in the affected semicircular canal) in children [5,40,54,55,56]. Furthermore, according to Ward et al. [55], electrocochleography can be utilized as a diagnostic test for TWSs (the elevated ratio of summation potential to action potential in the absence of SNHL). After a detailed history, combining the audiological outcomes with the findings of pediatric vestibular evaluation also provides clinicians with clearer evidence of the diagnosis with a holistic approach before radiological scans.

A fundamental difference between adult and pediatric phenotypes of TWSs is the difficulty in establishing the diagnosis by history. In young children, history is gleaned from parents and developmental milestones and guided by leading questions about routine pediatric activities [40]. This questioning demands holistic and dedicated pediatric training, and without this insight, diagnoses may be missed.

## 5. Conclusions

In this literature review, key points in the audio-vestibular evaluation of pediatric PCHL caused by TWSs were discussed considering the literature. The number of studies on third window syndrome in children is more limited than that in adults. Pediatric TWSs can be challenging to diagnose; thus, identifying the etiologic factors that can explain the disease is necessary. Through a holistic approach, using a comprehensive history and a complete pediatric audio-vestibular evaluation, TWSs causing PCHL in children can be easily detected early. This review provides more information to clinicians about the pitfalls of audio-vestibular evaluation in children with PCHL caused by TWSs.

## Figures and Tables

**Table 1:** Recent studies including pediatric population on audiovestibular evaluation and findings to differentiate third window syndromes.

Reference	Age Range (Year)	Number of Cases with TWSs	Etiology of TWSs	Audiological Tests	Audiological Findings	Vestibular Tests	Vestibular Findings
Dasgupta, et al. 2020 [5]	5–17	8/8	SCD, X linked, Multiple	Pure tone audiometry both AC and BC with masking (if necessary) Tympanometry Acoustic reflexes TEOAE	ABG at low frequency Normal tympanometry Mostly AR present Mostly TEOAE present	vHIT cVEMP at 500 Hz TB	Decreased VOR gain in the affected SSC and mostly saccades present Increased amplitude in the side of the lesion and usually decreased thresholds
Dasgupta, et al. 2019 [40]	5–17	13/580	SCD	Pure tone audiometry Tympanometry Acoustic reflexes TEOAE	Mixed, conductive, or SNHL Normal impedancemetry Mostly AR Present Mostly TEOAE Present	vHIT VNG Rotatory chair Vesibulo-spinal tests	Decreased VOR gain and presence of saccades Mostly normal oculomotor function Majority of normal rotatory chair Majority of normal vestibulospinal test
Kim, et al. 2021 [54]	12–80	60/60	PLF	Pure tone audiometry both AC and BC	The presence of ABG at low frequency in 45% of cases	Spontaneous nystagmus Positional test	The presence of spontaneous nystagmus in 35% of cases The presence of positional nystagmus in 92% of cases
Bonnard, et al. 2023 [46]	1–25	27	EVA, IP-II	Not available	Not available	vHIT cVEMP at 500 Hz TB Caloric test iced water	SSC hypofunction on the non-implanted side in 13.6% of patients Vestibular loss in otolith function on the non-implanted side in 13.3% of patients Canal loss on the non-implanted side in 13.6% of patients
Castellucci, et al. 2013 [35]	8–80	45	Unilateral SCD	Tympanometry Pure tone audiometry both AC and BC	An asymmetry between tympanometry peak compliance of the involved side Asymmetry ratio of compliance at the eardrum ≥ 14% in favor of the pathologic ear ABG > 20 dB nHL	Not available	Not available
Castellucci, et al. 2013 [38]	8–88	73	SCD	Pure tone audiometry both AC and BC Tympanometry	Low-frequency ABG Both AC PTA and ABG associated with the SCD size Type A tympanogram Insufficient association between peak tympanometry compliance and SCD location and size	AC and BC cVEMP and oVEMP at 500 Hz TB vHIT	Both amplitudes and thresholds of cVEMPs and oVEMPs associated with the SCD size Lower VOR gain in dehiscence at arcuate eminence than ampullary arm

TWSs: third window syndromes; AC: air conduction; BC: bone conduction; SCD: semicircular canal dehiscence; EVA: large vestibular aqueduct; IP-II: incomplete partition Type-II; PTA: pure tone average; PLF: perilymphatic fistula; vHIT: video head impulse test; VEMP: vestibular evoked myogenic potential; oVEMP: ocular VEMP; cVEMP: cervical VEMP; TB: tone burst; ABG: air-bone gap; VOR: vestibular–ocular reflex; TEOAE: transient evoked otoacoustic emission; SN: sensorineural; HL: hearing loss; VNG: videonystagmography; AR: acoustic reflex.
